# Supplementary material for: A framework for identifying the recent origins of mobile antibiotic resistance genes
Source: Commun Biol. 2021 Jan 4;4:8. doi: 10.1038/s42003-020-01545-5 (PMC7782503; doi:10.1038/s42003-020-01545-5)
Supplement: Supplementary file 2 — Supplementary Information [file 42003_2020_1545_MOESM2_ESM.pdf]

Supplementary Note 1

Aminoglycoside resistance genes

APH(3')-IV from Acinetobacter guillouiae A large scale screening for aminoglycoside resistance genes in *Acinetobacter spp* revealed the chromosome of A*. guillouiae* as the recent origin of the mobile *aphA6* gene, which was detected as part of composite transposon *TnaphA6* in other *Acinetobacter* spp, such as *A. baumannii*[1]. PCR-based analysis and WGS showed that the *aphA6* gene was present in the great majority of *A. guillouiae* isolates, whereas it was only sporadically detected in other *Acinetobacter* spp. High nucleotide identities between both coding and non-coding regions of *aphA6* positive MGEs and the *A. guillouiae* chromosome suggest that *aphA6* was mobilized from *A. guillouiae*, which was shown susceptible to amikacin. *ISAba125*, flanking *aphA6* in *TnaphA6*, provides a hybrid promoter driving the expression of *aphA6* in *A. baumannii,* and it was shown that amikacin resistance correlated with *aphA6* expression. The results of our own analysis are in agreement with these previously reported results. Though *A. guillouiae* is mostly associated with external environments, it has been, though rarely, isolated from human infection sites.

ACC(6')-Ih from Acinetobacter gyllenbergii The same screening for aminoglycoside resistance genes as described above revealed *Acinetobacter gyllenbergii* as the recent origin of *acc(6')-Ih*, a plasmid-borne gene associated with high level amikacin resistance[2]. The gene was found in all screened *A. gyllenbergii* isolates, where there genetic environment was identical over several thousand bp. On plasmid, the gene was flanked by IS (IS*Aba23*, IS*Ascp5*), but still retained >1kbp of its native genetic context, identical to the *A. gyllenbergii* chromosome. It was shown that *acc(6')-Ih* provided low level resistance in *A. gyllenbergii,* but high level resistance in *A. baumannii*. Expression of plasmid-borne *acc(6')-Ih* was driven by the IS*Aba23* provided promoter. However, the native and IS*Aba23* provided promoter were similarily potent, and the difference in resistance in *A. gyllenbergii* and *A. baumannii* was attributed to gene dosage. The results of our own analysis are in agreement with these

previously reported results. Though rare, *A. gyllenbergii* has been identified from clinical isolates.

Beta-lactam resistance genes

Class A beta-lactamases

SHV from Klebsiella pneumoniae SHV-type beta-lactamases are amongst the most common beta-lactam resistance determinants detected in *Enterobacteriaceae*[3]. A vast number of variants, many of them differing to others in just one amino acid, is known to date (SHV-1 to SHV-191, CARD 2019), found in a wide range of bacterial pathogens[4]. Using a comparative genomics approach including synteny analysis, it was shown that SHV-type enzymes were mobilized from the *Klebsiella pneumoniae* chromosome, where the gene is native[5], at least two times[6]. The two analyzed mobile SHV-variants, both associated with *IS26* but found on different MGEs, each retained several hundred base pairs of the chromosomal *K. pneumoniae* SHV locus. Though the authors presented evidence for *IS26* mediated mobilization, other SHV-variants are not associated with *IS26*, further suggesting several separate mobilization events. That said, some SHV-variants are probably the result of post-mobilization mutation. These results complied with the results of a previous study, which used molecular approaches to identify *K. pneumoniae* as the origin of SHV-type beta-lactamases[7]. The results of our own analysis are in agreement with these previously reported results. *K. pneumoniae* is an important bacterial human pathogens, and frequently associated with multidrug resistance and high mortality rate[8]. However, *K. pneumoniae* is also associated with external environments, such as soil or surface water samples.

CTX-M from Kluyvera spp Due to their potency, their global spread and broad host range, the >50 reported CTX-M enzymes are among the most investigated antibiotic resistance enzymes. There is a detailed review discussing their spread and origin[9], making a detailed description in this article unnecessary. In short, it has been shown that different CTX-M variants

have been mobilized from different *Kluyvera* spp, mainly *K. ascorbata* and *K. georgiana*[10–12] (until now), based on sequence similarities and detailed comparison of the CTX-M genetic environments on both MGEs and the *Kluyvera* chromosome. Most commonly, CTX-M enzymes are associated with *ISEcp1* and *ISCR1* on MGEs, both of which provide a promoter that drives expression of the beta-lactamase. In their native *Kluyvera* context, CTX-M enzymes are only weakly expressed[9] and usually do not confer high-level resistance. There is strong evidence that the different CTX-M clusters, and even variants within the same cluster, have been mobilized independently through involvement of different *IS*/*IS*CR elements[10–14]. The results of our own analysis are in agreement with these previously reported results. *Kluyvera* spp. are relatively rarely isolated from human clinical samples, but little is known about where members of that genus thrive otherwise.

PER from Pararheinheimera spp Large scale comparative genomic analysis of PER-type beta-lactamase-containing genomes from public databases revealed the genus *Pararheinheimera* as the origin of mobile PER-type genes, based on conserved synteny and high nucleotide identities of up to 96% between PER positive MGEs and the chromosomal *Pararheinheimera* PER locus[15], including not only the PER-gene, but also several other genes. Mobile PER genes were associated with different *IS/IS*CR elements, such as ISCR1 (often in chinease isolates[16]) and *ISPa*12/*ISPa13*, the latter two forming a composite transposon in some cases able to mobilize and drive expression of the PER gene via an IS*Pa12* provided promoter[17]. There is much evidence suggesting that different PER genes have been mobilized independently from one another, in different parts of the world. Not all analyzed *Pararheinheimera* isolates carried PER-like genes, indicating that these genes were acquired at some point of the genus' evolutionary history. This origin was identified through our comparative genomics workflow. Little is known about the genus *Pararheinheimera*. PER-positive isolates have been isolated from soil-, saline- and freshwater environments, but have not yet been reported in association with disease[18,19].

KPC – orthologues in Chromobacterium spp Enzymes with relatively high (from a long term evolutionary perspective) amino acid identity to KPC-2 (up to 76%) were detected without presence of MGEs in different *Chromobacterium* spp[20]. Due to this, compared to other origin reports, low identity of the *Chromobacterium* KPC-like gene towards mobile KPC-variants, it is unlikely that one of the examined *Chromobacterium* strains is the source of mobile KPC. Our own analysis identified KPC orthologues in other *Chromobacterium* isolates as well, though with a maximum amino acid identity of 78%. Though it is possible that to date undescribed/undetected *Chromobacterium spp* or a closely related genus is/are the source of mobile KPC, further research would be needed to investigate this hypothesis.

***GPC-1/BKC-1 from Shinella* spp.** Two novel class A carbapenemases have been recently characterized, sharing 77% amino acid identity[21,22]. Genome analysis in a recent study showed the presence of GPC-1/BKC-1-like genes identified in *Shinella* species, displaying maximum nucleotide identities from 87-89% to the mobile GPC-1/BKC-1 like genes[23]. On plasmid, GPC-1 was found associated with *IS91* and a *tnpA* gene, whereas BKC-1 was associated with IS*Kpn23*. No IS were identified at the *Shinella* GPC-1/BKC-1 locus, and no other genes at the site indicated mobility. Different *Shinella* spp. furthermore display conserved synteny at the locus. Therefore, both carbapenemases were likely mobilized from to date unsequenced *Shinella* spp, though we cannot (due to the relatively low nucleotide identity) exclude that the genes have been mobilized from a genus closely related to *Shinella.* Different *Shinella* species have been isolated from mainly soil samples, and not been reported to be involved in infection to date.

Class B beta-lactamases

LMB-1 from Rheinheimera pacifica A novel subgroup B3 Metallo-Beta-Lactamase (MBL), LMB-1, isolated from an *Enterobacter cloacae* plasmid, was described recently. A search for the MBL encoding gene in GenBank showed that the gene was 99% identical to a gene located on a *Rheinheimera pacifica* contig. Further analysis of both the plasmid sequence and the *R.*

pacifica contig revealed the same gene upstream of LMB-1 in both cases (though truncated on plasmid), though the MGEs located up- and downstream of plasmid-borne LMB-1, IS6- and IS91-family transposases, were missing on the *R. pacifica* contig. The detection of housekeeping genes on the *R. pacifica* contig supports the assumption that the contig is part of the chromosome of this species. Our own analysis identified LMB-1 in other *Rheinheimera* isolates (*R. nanhaiensis, Rheinheimera* sp.). However, nucleotide identities of these isolates were only about 80% compared to the *R. pacifica* contig, but the synteny at the locus appears somewhat conserved. Though the lack of LMB-1 positive genomes makes it difficult to draw a definitive conclusion and more LMB-1 containing genomes are needed, our data support the notion that LMB-1 may have been mobilized from *R. pacifica*, a species associated with marine environments[24].

Class C beta-lactamases

FOX, CMY-1/MOX from Aeromonas spp It had been suspected for some time that the mobile FOX and CMY-1/MOX cephalosporinases were mobilized from the *Aeromonas* chromosome, as they displayed similarity to the chromosomal *AmpC* of several *Aeromonas* spp[3]. Large scale genomic analysis revealed that these genes have been mobilized from at least four different *Aeromonas* spp: FOX-type genes from *A. allosaccharophila*[25]*,* CMY-1/MOX-1 from *A. sanarelii,* MOX-2 from *A. caviae* and *MOX-9* from *A. media*[26]. In most cases, fractions of the *Aeromonas AmpC*-locus have been mobilized together with the *ampC* gene, with nucleotide identities ≥98% between mobile loci and the respective *Aeromonas ampC*-locus. FOX-type enzymes where associated with different types of IS, such as IS26, ISAs2 (as composite transposon) or a Tn3-like structure. It is possible that association of different FOX-variants with different *IS*/transposons represent separate mobilization events, as supported by different spacer lengths between the respective IS and the cephalosprinase gene. The analyzed CMY-1/MOX variants were associated with *ISCR1* or *ISKpn*9. Though it has not been shown experimentally, their expression may be driven by IS/ISCR1, which has been shown to contain an outward oriented promoter driving the expression of adjacent genes[27]. These origins were

identified through our comparative genomics workflow, correcting a previous report which suggested *A. caviae* as the origin of FOX-type genes. Aeromonads thrive in aquatic environments and are known to cause infections in humans and animals, and reports of *Aeromonas* infection have increased during the last decade[28,29].

CMY-2 from Citrobacter spp CMY-2-type AmpC beta-lactamases are the most commonly reported cephalosporin resistance determinants in *Enterobacteriaceae*[30]. Shortly after the first reports of mobile *AmpC* cephalosporinases emerged, it was noted that mobile CMY-2-type enzymes were highly similar to the chromosomal *Citrobacter freundii* AmpC. Subsequent comparison of the genetic environments of both mobile CMY-2-like genes and the chromosomal *C. freundii ampC* genes showed that the order and orientation of genes downstream of the *AmpC* were identical, and shared >97% nucleotide identity[31]. Mobile CMY-2-like genes were always associated with *ISEcp1*, and it is likely that this IS is responsible for the genes mobilization, as *ISEcp1*-mediated mobilization of CMY-2 from the *C. freundii* chromosome has recently been shown in vitro[32]. The results of our comparative genomics analyses were in agreement with the previously reported results. Association of CMY-2 with *ISEcp1* and a multi-copy plasmid was shown to significantly increase resistance to cephalosporins. *C. freundii* can be found in environmental samples for e.g soil or water, but is also frequently associated with disease, especially in immunocompromised patients.

DHA from Morganella morganii As the plasmid-borne DHA-1 cephalosporinase was discovered in a *Salmonella enteriditis* isolate, it was noted that the enzymes amino acid sequence was highly similar to that of the chromosomal *Morganella morganii AmpC*. Sequence analysis showed that not only the *ampC* gene, but also the upstream region, containing an *ampR* gene as well as about 110bp of non-coding DNA, were ≥97% similar to the chromosomal *M. morganii ampC* locus. Several other genes associated with the chromosomal *M. morganii* DHA-locus were also present on DHA-positive MGEs. Expression of DHA-type *AmpC*s on plasmids does not seem to be regulated by MGE-provided promotors, but by the *ampR* gene located upstream of DHA, as in the genes native *M. morganii* context[33–35]. The results of our

comparative genomics analyses were in agreement with the previously reported results *M. morganii* is an enteric bacterium found in mammals, and as opportunistic pathogen mostly causes wound any urinary tract infections[36].

ACT from Enterobacter spp To date, 38 ACT-variants have been reported in CARD (August 2019), most of them in *Enterobacter* spp. Previously thought to have originated in *Enterobacter cloacae*, it was later shown that the mobile ACT-1 *AmpC* most likely originated in *Enterobacter asburiae*. Whereas the *E. cloacae ampC and ampR* genes were only 85-91% similar to the chromosomal *ampC* locus in *E. cloacae*[37], they were ≥95% identical in nucleotide identity to the chromosomal *ampC*-locus of *E. asburiae*[38]. ACT-1 expression seems to be regulated by *ampR,* on both the *E. asburiae* chromosome and MGEs. Other mobile ACT-variants display greater similarity to the chromosomal *AmpC*-locus of other *Enterobacter* spp, such as *E. hormaechei* and *Enterobacter kobei*, which however belong to *Enterobacter cloacae* complex[39], and are as such also referred to as *E. cloacae.* It is thus possible that different variants are the result of separate mobilization events from different *Enterobacter* spp from this complex. The results of our comparative genomics analyses were in agreement with the previously reported results. *E. asburiae* is an opportunistic pathogen that has repeatedly been isolated from human clinical specimen[40], but is also found in soil and water.

MIR from Enterobacter cloacae When the mobile MIR-1 AmpC was first reported in 1990 from clinical *Klebsiella pneumoniae* isolates, it was noted that its' gene was more similar (about 90% nucleotide identity) to the *ampC* gene of *Enterobacter cloacae* then to the *ampC* gene of other *Enterobacteriaceae*. This led to the hypothesis that mobile MIR-1 have been mobilized from the *Enterobacter cloacae* genome, though critical evidence was lacking due to the relatively low sequence similarity[41,42]. Later, another study reported an *ampC* of an *E. cloacae* isolate from a clinical sample which was 98% similar to MIR-1. I-CeuI restriction followed by by hybridization of the obtained fragments with probes for *ampC* and 23S rRNA confirmed the chromosomal location of the MIR-1-like *ampC* gene[43]. To date, 18 variants of MIR have been

reported, most of them in *Enterobacter* spp. The high level expression of MIR-1 is likely driven by a hybrid promoter formed during the genes mobilization[44].

ACC from Hafnia alvei Due to it's for *AmpC* enzymes unusual resistance type, unaffected susceptibility to cefoxitin, the chromosomal *Hafnia alvei AmpC* was identified as the origin of plasmid-borne ACC-type *AmpC* cephalosporinases. Sequence analysis showed that the chromosomal *H. alvei AmpC* and plasmid-borne ACC-1 were ≥99% identical[45]. Further analysis revealed that the *gdhA* gene detected downstream of mobile ACC-1 is also present at the *H. alvei AmpC*-locus. To date, five ACC-like enzymes have been reported, though only two (ACC-1 and ACC-4) in other species than *H. alvei.* ACC-4 differs from ACC-1 only by a point mutation[46], and extensive similarities in the genetic environment of ACC-1 and ACC-4 suggest that ACC-4 is a product of post-mobilization evolution. In non-*H. alvei* isolates, ACC-like genes are preceded by *ISEcp1*. As the native *ampR* regulator gene is missing on these MGEs, it is likely that *AmpC* expression is driven by *ISEcp1* for ACC-1, as shown for other resistance genes associated with this IS[12,47]. On the described ACC-4 MGE, *ISECp1* was however truncated by IS26. The results of our comparative genomics analyses were in agreement with the previously reported results. Hafnia alvei is found on plants as well as in the mammalian gastrointestinal tract, but also has been shown to be an opportunistic human pathogen[48].

Class D beta-lactamases

OXA-23 from Acinetobacter radioresistens OXA-23 provides carbapenem resistance, and has been reported mainly in resistant *Acinetobacter baumannii* isolates. Due to the genes GC content and it's prevalence in *A. baumannii*, the genes were suspected to originate in some *Acinetobacter* species. This ultimately led to the identification of *A. radioresistens* as the source of mobile OXA-23 genes. OXA-23 like genes with high identity towards mobile OXA-23 were identified in all investigated *A. radioresistens* strains. Hybrididization of OXA-23- and 16S rRNA

probes on the same fragment following I-CeuI restriction as well as the absence of *ISAba1* and *ISAba4*, with which mobile OXA-23 is usually associated, showed that the gene was located on the *A. radioresistens* chromosome and suggested non-mobility. The gene encoded downstream of OXA-23 in its mobile context, coding for an AAA Atpase, was also detected with high identity to its mobile counterpart downstream of the OXA-23-like genes in *A. radioresistens*. However, *A. radioresistens* was shown to be fully susceptible to carbapenems, suggesting that the native OXA-23 gene is expressed at low levels or not at all[49]. It had been shown previously that mobile OXA-23 genes were expressed through promoters provided by the respective associated IS, either IS*Aba1* or IS*Aba4*[50]. The results of our comparative genomics analyses were in agreement with the previously reported results. *A. radioresistens* is part of the commensal skin flora and has been reported, though rarely, to be involved in infection in immunocompromised patients[51].

OXA-181 from Shewanella xiamenensis OXA-181 is a OXA-48-like, mobile carbapenemase first identified in *K. pneumoniae*. Though OXA-48 like carbapenemases hydrolize carbapenems less efficiently than other carbapenemases, they have nevertheless spread globally during the last years. Several closely related variants have been identified (OXA-161, OXA-163, OXA-181, OXA-199, OXA-204, OXA-232, OXA-244, OXA-245, OXA-247, OXA-370, OXA-405 and OXA-416). In 2011, *Shewanella xiamenensis* was shown to be the origin of OXA-181, which was first detected in a *Klebsiella* isolate, using a PCR and sequencing based approach for screening of environmental samples. Sequencing revealed that the genetic environment of both mobile OXA-181 and the OXA-181 gene on the *S. xiamenensis* chromosome were identical, except for the presence of *ISEcp1*, which is associated with mobile OXA-181[52] and likely drives its expression[53]. Recently, a large scale screening of *Shewanella* spp for OXA-48-like genes, both *in vivo* and *in silico*, showed the presence of those genes in a conserved context on the chromosomes of several *Shewanella* spp[54]. This also indicates that mobilization of different OXA-48-like genes may have happened

independently several times, from different *Shewanella* spp. The results of our comparative genomics analyses were in agreement with the previously reported results. Some *Shewanella* spp are known opportunistic pathogens, and reports of *Shewanella* caused infections have increased during the last years.

OXA-51-like from Acinetobacter baumannii Carbapenem resistance in *Acinetobacter baumannii* is often due to chromosomally encoded carbapenemases[55], such as OXA-51-like enzymes, whose expression is upregulated through e.g promotors provided by IS. OXA-51-like genes are instrinsic to *A. baumannii,* However, plasmid-borne OXA-51-like genes in combination with *ISAba1*, an IS associated with OXA-51-like overexpression, have been reported. Using a PCR-based approach, it was shown that the plasmid-associated OXA-51 region was most likely mobilized from the *A. baumannii* chromosome via a one ended transposition mechanism. The genes encoded downstream of both plasmid- and chromosome encoded OXA-51 were found to be identical. Whereas the authors did not exclude that plasmid borne OXA-51-like-loci (such as the ones containing OXA-82 and OXA-172) may have emerged from different mobilization events, other plasmid-borne OXA-51-like enzymes were suggested to be the consequence of mutation of already plasmid-borne OXA-82 and OXA-171. It was also noted that not all *A. baumannii* isolates in which *ISAba1* preceded *OXA-51*-like genes displayed resistance to carbapenems, and increased gene dosage of high copy number OXA-51-like positive plasmids was suggested as possible explanation for resistance. The results of our comparative genomics analyses were in agreement with the previously reported results. *Acinetobacter baumannii* is a common cause of hospital acquired infection in humans, associated with high mortality rates[56].

Colistin resistance genes

MCR-2 from Moraxella pluranimalium Plasmid-borne MCR genes providing resistance to polymyxin have been identified relatively recently. Several plasmid borne variants have been

discovered since then, with sequence divergences >50% between some variants. This degree of sequence divergence alone strongly suggests that different MCR variants were mobilized from different origins. Genes related to MCR-2 were discovered on the chromosomes of *Moraxella* spp[57], and later, using a PCR-based approach, it was shown that *Moraxella pluranimalium* contains a most likely chromosomal MCR gene that is 99% identical to MCR-2. Attempts to extract plasmids or transfer polymyxin resistance from *M. pluranimalium* to *E. coli* failed, indicating that the gene is indeed non-mobile in *M. pluranimalium.* Furthermore, the gene found downstream of mcr-2 in *M. pluranimalium* is also found at the mobile MCR-2 locus, with high nucleotide identity. Mobile MCR-2 is associated with IS1595, which may have mobilized the gene from the *M. pluranimalum*[58] chromosome. The results of our comparative genomics analyses were in agreement with the previously reported results. *M. pluranimalium* has been isolated mainly from pigs, both healthy and diseased, but little is known about the species otherwise.

MCR-3 from *Aeromonas spp*

Though not explicitly claimed, there is some evidence that MCR-3, a mobile MCR-gene that is 47% identical to MCR-2, may have originated in *Aeromonas* spp., as its sequence was found 74-95% to MCR-like genes found in several *Aeromonas* spp[59]. Sequence analysis showed that the gene encoded downstream of mobile MCR-3 is also found in MCR-3 positive Aeromonas, though the gene arrangement at the MCR-3 like locus differs slightly from the mobile MCR-3-like locus in all up to date analyzed *Aeromonas* genomes (though it is similar between many *Aeromonas* spp.)[60]. Further research is needed to provide a definite answer to whether MCR-3 genes originated in *Aeromonas* spp, as indicated by previous reports.

MCR-4 from *Shewanella frigidimarina* The finding of the mcr-4 gene, 100% identical to mcr-4.3, on the chromosome of *Shewanella frigidimarina* incited the authors of the study to hypothesize about the origin of MCR-4.3 in *S. frigidimarina*. However, genes associated with mobility (Tn5044) and antibiotic resistance (TetR) are encoded directly downstream of the

MCR-4.3 gene[61], and the aligning region with the mobile MCR-4 locus only encompasses the MCR gene[62]. Analysis of the genetic environment of mcr-4 gene variants in *Shewanella* spp showed the presence of multiple MGEs at the mcr-4 locus, suggesting that these genes have been acquired. The evidence for *S. frigidimarina* as the origin of mobile MCR-4 is thus weak. However, Proteins related to MCR-4 are found on the chromosomes of several *Shewanella* spp. with up to 80% amino acid similarity, suggesting, though critical evidence is lacking, that these genes may have been mobilized from some to date unsequenced Shewanella spp.

MCR-8 from *Stenotrophomonas* spp

A recent study suggested *Stenotrophomonas* spp as the origin of mobile mcr-8 genes, based on the identification of MCR-8 homologues in *Stenotrophomonas* genomes[63]. However, the respective homologues in the genomes of *S. maltophilia* and *S. rhizophila* are at maximum 63% identical at amino acid level to mobile MCR-8. Though it is possible that to date unknown/unsequenced *Stenotrophomonas* sp. harbor MCR-8 enzymes more closely related to mobile MCR-8, there is no evidence for an emergence of mobile MCR-8 from the *Stenotrophomonas* genome. Due to the low sequence similarities, the MCR-8 homologues in *Stenotrophomonas* were not detected in our analysis, making it at the same time unlikely that these genes were mobilized from *Stenotrophomonas* recently, as appears to be the case for all other IS-associated ARGs described in this study.

MCR-9 from *Buttiauxella* spp

A novel plasmid-borne *mcr* variant, named *mcr-9*, was isolated from colistin resistant *E. coli*. *In silico* identified the gene in several other enterobacterial species, such as *K. pneumonia* and *Enterobacter* spp. Associations with several different IS was described, as well as variations in the genetic context downstream of the gene. In all cases *IS903*-like elements were observed upstream of the gene, followed by *mcr-9* and the gene *wbuC*[64]*.* This context is either followed by an IS*26*-like element, or the genes *qseC* and *qseB*, a two component regulatory system

that may play a role in inducing *mcr-9*. The authors furthermore identified *mcr-9*-like genes in several *Buttiauxella* spp. isolates, with up to 84% amino acid identity between the plasmid-borne gene and the *mcr-9-like* genes in *Buttiauxella* spp. The gene *wbuC* was encoded downstream of the *mcr-9* like genes in *Buttiauxella* spp., but the two-component regulatory system was missing, and it was speculated that these genes were derived from another source. Our comparative genomics analysis identified *mcr-9*-like genes in 13 *Buttiauxella* spp. isolates. Comparison to the mobile *mcr-9* from a *Salmonella enterica* plasmid (carrying the *mcr-9,wbuC,qseC,qseB* genes) showed that five isolates carried the *wbuC* gene downsteam of the *mcr-9*-like gene, but lacked IS*903*. The *mcr-9*-like gene and *wbuC* were 77-83% similar to the plasmid-borne *mcr-9* and *wbuC* genes over 2178bp. While this suggest some taxonomic relation to the origin, the low nucleotide identity suggests that these genes may have been mobilized from another genus as well. And indeed, we identified several *Leclercia adecarboxylata* genomes carrying *mcr-9*-like genes without any IS in their genetic environment, displaying up to 93% amino acid identity to the mobile MCR-9 protein. Furthermore, these *L. adecarboxylata* isolates encoded both *wbuC* and *qseC/qseB* downstream of the *mcr-9*-like gene. The nucleotide identity to the mobile *S. enterica* locus was 85% over 4531bp. The identity of the *L. carboxylate mcr-9*-like locus to only the mobile *mcr-9* and *wbuC* genes was 88% over 2411bp. That said, this *mcr*-9 like locus was not found in the majority of *L. adecarboxylata* genomes, which may suggest that the locus was acquired by certain species/lineages of *Leclercia*. Thus, the origin of MCR-9 is likely more closely related to *L. adecarboxylata* than to *Buttiauxella* spp., but further research and genomic data are needed to pinpoint the origin of MCR-9.

Fluoroquinolone resistance genes

QnrA from Shewanella algae *QnrA* genes where identified in several *Shewanella algae* isolates through screening several genera of clinically significant bacteria using PCR.

Subsequent analysis revealed the location of *qnrA* on the *S. algae* chromosome, whereas ISCR1 (called *orf513* at that time), the MGE associated with plasmid-borne *qnrA* and likely involved in the genes mobilization and high level expression, could not be detected[65]. Other reports of *S. algae* containing chromosome-borne *qnrA* genes[66] as well as our genomic synteny based analysis of *qnrA* containing replicons support *S. algae* as the origin of mobile *qnrA.* Though displaying higher fluoroquinolone MICs than other, *qnrA*-negative Shewanellaceae, *S. algae* was susceptible to fluoroquniolones. S. *algae* thrives in aquatic environments (both fresh- and saline water) and may cause disease upon ingestion of contaminated sources such as water or shellfish. The results of our comparative genomics analyses were in agreement with the previously reported results. Reports of *S. algae* infection have increased during the last decades, especially in Asia during the summer months[67]. In *S. algae*, *qnrA* seems to be involved in thermoregulation[66], and *S. algae* has been shown to be susceptible to fluoroquinolones[65].

QnrB from Citrobacter spp Based on the relatively high prevalence of *qnrB* alleles in *Citrobacter* spp, it was hypothesized that the mobile *qnrB* alleles were mobilized from the *Citrobacter* chromosome. A study of >70 clinical *Citrobacter* isolates, targeting *qnrB* alleles by PCR, indicated that *qnrB* alleles were mostly present in isolates belonging to the *C. freundii* complex, including *C. freundii, C. braakii, C. youngae* and *C. werkmanii*. Some *qnrB* alleles were specific to certain subgroubs, e.g *qnrB27* was only found in *C. braakii* isolates. I-CeuI restriction followed by double hybridization with *qnrB* and 23S rRNA genes showed that *qnrB* alleles were located on the chromosome of most isolates. Two isolates were shown to harbor plasmid-borne *qnrB* genes. PCR for *ISCR1* and several integrase genes, which are associated with mobile *qnrB* genes, was negative except for the isolates harboring the *qnrB*-positive plasmids and transfer experiments failed for all isolates except for the plasmid-carrying ones. Sequence analysis showed that the synteny between the chromosomal *Citrobacter* spp. *qnrB* locus and plasmid-borne *qnrB* loci was identical, and nucleotide identities between the two loci were high. These results strongly suggest that mobile *qnrB* genes were mobilized from the

chromosome of members of the *C. freundii* cluster[68]. A more recent study managed to establish associations between eight varying chromosomal *Citrobacter* spp *qnrB* loci and plasmid borne *qnrB* genes, further suggesting multiple mobilization events[69]. QnrB positive *Citrobacter* isolates were mostly susceptible to fluoroquinolones, which was also shown recently in another study[70]. As shown for the origins of several other MARGs, not all members of the respective genus carried *qnrB*-alleles, indicating that these genes must have been acquired, or lost, in some strains during their evolutionary history. The results of our comparative genomics analyses were in agreement with the previously reported results. Interestingly, though *qnrB*-like genes are associated with *ISEcp1* and IS*CR1*, both of which are known to be able to increase expression of adjacent genes, it was shown for two *qnrB* variants, *qnrB2* (associated with ISCR1) and *qnrB19* (associated with IS*Ecp1*) that *qnr* expression is not driven by these IS, but regulated (at least for *qnrB2*) in a *lexA* dependent manner, and thus inducible by the bacterial SOS response[71,72]. *C. freundii* is an opportunistic pathogen increasingly reported in nosocomial infections, but is also a common commensal of the human gut. It is also found in environmental samples, such as soil and water[73].

***QnrE* from Enterobacter spp** Low-level fluoroquinolone resistance in a clinical *K. pneumoniae* isolate ultimately revealed the presence of a novel, plasmid-borne *qnr* gene, named *qnrE1* in that isolate. Sequencing of the plasmid harboring the gene revealed that *qnrE1*, 73-76% similar to previously described *qnr* genes, was preceded by IS*Ecp1* encoded closely upstream of the gene. Comparative genomic analysis showed that the *qnrE1* containing region, including several downstream genes (although partly truncated), were highly identical to the chromosomes of five *Enterobacter* spp. genomes (2 *E. cloacae* and 2 *E. asburiae*), with nucleotide identities ranging from 83-95%. IS*Ecp1* was not detected upstream of *qnrE1* in these isolates. As IS*Ecp1*-mediated mobilization of resistance genes has been repeatedly shown[13,32,47], it was suspected that IS*Ecp1* mobilized *qnrE1* from the *Enterobacter* chromosome and is involved in expression of plasmid mediated *qnrE1*. Differential spacer lengths between the IS and *qnrE* on different plasmids suggest separate mobilization events[74].

That *qnrE1* was not found on all *Enterobacter spp.* chromosomes mirrors what was previously observed for other MARGs, such as PER-type genes and *qnrB* genes, and suggests that *qnrE1* was acquired by some *Enterobacter spp* strains during their evolutionary history. Our own comparative analysis showed the mobile *qnrE1* locus was, from all *Enterobacter* spp*,* consistently most similar to the *Enterobacter mori qnrE1* locus, sharing identities 98-≥99% over 2-3 kbp (including co-mobilized genes), making this species the most likely origin of mobile *qnrE* genes. *E. cloacae, E. asburiae*, and *E. mori,* on whichs chromosomes *qnrE* homologs were identified, are opportunistic pathogens of humans and other animals.

QnrS from Vibrio splendidus Though an article titled '*Vibrio splendidus* as the source of plasmid-mediated QnrS-like quinolone resistance determinants ', describes the presence of QnrS-like genes in *Vibrio splendidus* (up to 87.6% identical in amino acid sequence to QnrS1 and QnrS2) and other *Vibrio* spp. (maximum 64% identical to QnrS1 and QnrS2). Large scale genome analysis confirms the presence of *qnrS* like genes in *V. splendidus* and other species of *Vibrio*, though at ≤90% amino acid identity. Though our examination of the genetic environment of *qnrS* in *V. splendidus* suggests that the gene is non-mobile there and presence of chromosomal *qnrS* genes in other Vibrionaceae indicates that these genes may have been mobilized from some *Vibrio* or closely related *Photobacterium* spp, there is no strong evidence for *V. splendidus* as the origin of mobile QnrS genes.

OqxAB from Klebsiella pneumoniae The plasmid-encoded multidrug efflux pump OqxAB was first detected in swine-derived isolates from northern Europe, and can, among resistance to olaquindox, which is used in industrial swine-growth-promotion, confer resistance to fluoroquinolone antibiotics. It is encoded by two separate genes, *oqxA* and *oqxB,* which are chromosomal in *Klebsiella pneumoniae*, based on studies using PCR and hybridization approaches. Using PCR, a large collection of clinical isolates was screened for the presence of *OqxAB*. The great majority of *OqxAB*-positive positive isolates were *K. pneumoniae*, with only a few *OqxAB*-positive *E. cloacae* and one *E. coli*. Sequence comparison showed that the *OqxAB* sequence from *E. coli* displayed ≥98% sequence similarity with the chromosomally

located *K. pneumoniae oqxAB* genes. DNA hybridization showed that the *oqxAB* genes from *E. coli* were located on a large plasmid, whereas *oqxAB* probes hybridized with chromosomal DNA in *K. pneumoniae*, reaffirming the genes' chromosomal location. MICs for ciprofloxacin and olaquindox varied in *oqxAB* positive *K. pneumoniae*, suggesting the presence of additional resistance mutations in some strains. Sequencing showed that the plasmid-borne *oqxAB* genes in *E. coli* were flanked by *IS26*-like sequences, confirming their location in a transposon termed Tn*6010*[75]. The results of our comparative genomics analyses were in agreement with the previously reported results. As previously mentioned, *K. pneumoniae* is a human pathogen but also associated with external environments.

Fosfomycin resistance genes

FosA1 from Enterobacter cloacae In a study on the distribution of *fosA* genes in gram negatives, *Enterobacter cloacae* was proposed as the origin of plasmid-mediated *fosA1* genes[76]. *fosA1* (previously called *fosA,* designated *fosA1* here based on Ito et al 2017) was originally reported on transposon Tn*2921* found in *Serratia marcescens*. Though close relatedness between the Tn*2921* encoded *fosA1* and a *fosA1* gene encoded on the *E. cloacae* chromosome was stated, and the gene is present in nearly all available *Enterobacter cloacae* genomes, no detailed genomic analysis of the locus was performed. Our comparative genomic analysis showed that the *fosA1/fosA2* (the chromosomal *Enterobacter fosA* gene was termed *fosA2*) locus is present in several *Enterobacter* spp. While the genes in the immediate vincinity of the *fosA1/fosA2* gene are conserved, the genes sourrounding the locus display significant plasticity. At a 70% cutoff, *fosA1/fosA2-like* genes are present in roughly half of the *Enterobacter* spp. genomes in Genbanks Assembly database. Comparison to Tn*2921* shows high identity (max 98%) over 4631 basepairs to the *Enterobacter* spp. *fosA2*-locus. The alignment includes four (partially truncated) genes from the *Enterobacter* spp. locus and is flanked by IS4 (IS4 is absent from the *Enterobacter* spp. *FosA* locus). This suggests that the *fosA1* gene of Tn*2921* has been mobilized from an *Enterobacter spp* chromosome. However, there is no clear nucleotide identity 'gradient' across *Enterobacter* species that would allow an

assignment of the Tn2921-borne *fosA1* to a single species. Thus, while *E. cloacae* and *E. hormaechei* consistently harbor *fosA2* genes with high similarity to Tn2921-borne *fosA1*, it is difficult to pinpoint a single *Enterobacter* spp. As the origin of *fosA1* at the time of writing.

FosA3/4 from Kluyvera georgiana Two separate studies identified *K. georgiana* as the origin of the mobile fosfomycin resistance genes *fosA3* and *fosA4*, using PCR- and WGS-based (Whole genome sequencing) approaches. *fosA3* and parts of its genetic environment were found to be 94- 99% identical in nucleotide sequence to the chromosomal *FosA3*-like locus of two different *K. georgiana* genomes. The alignment started and stopped at the *IS26*-locus of the plasmid-borne *fosA3*-loci*,* which flank the locus on either side. The synteny at the *fosA3*-locus in both *K. georgiana* isolates was conserved, and the nucleotide identities differed around 5%. The authors also noted differences in the positioning of *IS26* relative to the *fosA3* gene on different *E. coli* plasmids, suggesting separate mobilization events of the different plasmid associated *fosA3*-loci. While plasmid-borne *fosA3* significantly increased fosfomycin resistance, both *K. georgiana* strains were susceptible to fosfomycin[77]. However, the *fosA3 gene* of the *K. georgiana* strain whichs *fosA3*-like-locus was shown to be the origin of mobile *fosA3,* was shown to be only 93% similar to mobile *fosA4* from a *Salmonella* spp plasmid. The genetic environment of mobile *fosA4*, including the gene itself and some neighboring genes, were shown to be >99% similar to the chromosomal *fosA4*-like locus of a *K. georgiana* isolate obtained from a bloodstream infection. The plasmid-borne *FosA4* was shown to be flanked by IS6 family transposases on both sides[78]. Both studies reported highly similar genetic environments of the *fosA* genes on the respective *K. georgiana* genomes. The divergence in nucleotide sequence of *fosA3* and *fosA4* genes, as well as their association with different IS and plasmids strongly suggests that they have been mobilized independently from different *K. georgiana* genomes. The results of our comparative genomics analyses were in agreement with the previously reported results

FosA5/6 from Klebsiella pneumoniae In two separate studies, fosfonamide resistant *E. coli* were isolated from patients. Both fosfomycin resistance determinants were carried on plasmids, sharing 96% amino acid identity, but only around 79% amino acid identity with previously described *fosA3*. The two novel resistance genes, shown to encode fosfomycin modifying enzymes, were termed *fosA5* and *fosA6*. The genes and their genetic environment were sequenced and subjected to comparative genomic analysis, revealing nucleotide similarities of 97->99% between *fosA5/6* and a glutathione transferase encoded on the chromosome of different *Klebsiella pneumoniae* isolates. Analysis of the genetic environment of both genes revealed two similar, but not identical plasmid-borne *fosA5/6*-loci. While *IS10* was located upstream of all mobile *fosA5/6*-loci, the length of spacer sequences between IS10 and *fosA5/6* differed. On two plasmids (pKP96, pHKU1), *fosA5* was flanked by *IS10* on both sides, whereas other IS (IS1, IS26) were encoded downstream of the *fosA5*-containing fragment on other plasmids (pHS33, pYD786). The presence of IS10 upstream of the *fosA5/6* containing fragment suggests a role of this IS in the genes' mobilization. However, the fragments of genes mobilized together with *fosA5/6* also differed between plasmids encoding *fosA5* and *fosA6*. While all *fosA5/6*-positive plasmids encode a truncated *lysR* transcriptional regulator between IS10 and the respective *fosA* gene (though the lengths of the truncated *lysR* differ), different truncated genes are encoded downstream of *fosA* on *fosA5* and *fosA6* plasmids. The genetic environments on the different plasmids were however ≥98% identical to the chromosomal *fosA*-like loci of different *K. pneumoniae* strains, strongly suggesting that the *K. pneumoniae* chromosome is the origin of these mobilized fragments. The results of our comparative genomics analyses were in agreement with the previously reported results. Since *lysR* is truncated in all plasmid-borne fragments, it is possible that *fosA* expression is driven by IS10, though further research is needed to investigate this hypothesis.

FosA8* from *Leclercia adecarboxylata

A fosfomycin resistant *E. coli* isolate was obtained from human urine. Whole genome sequencing of the strain led to the identification of a novel *fosA* gene, named *fosA8,* with 66-79% amino acid identity towards previously described *fosA* genes. The gene was located on a plasmid and inserted into the *sprT* gene. Though no IS or other transposases were described in direct vincinity of the gene, identical direct repeats on both sides of the gene strongly suggest the involvement of such an element in the acquisition of *FosA8*. A search against GenBank led to the identification of a highly similar (98% amino acid identity) FosA protein in *Leclercia adecarboxylata*. The sequences directly adjacent to the *fosA8* gene were 99% similar to those in *Leclercia adecarboxylata* in several isolates[79]. Our comparative genomic analysis supports the conclusions of the original report. We find that the *fosA8* locus in *Leclercia adecarboxylata* and other *Leclercia* spp (n=86) isolates lacks mobile genetic elements and has a conserved synteny, while nucleotide identities of the encoded genes differ up to >10% between different strains of *L. adecarboxylata*. The truncated, co-mobilized genes adjacent to *fosA8* on the originally reported *E. coli* plasmid are present in full length in the *L. adecarboxylata* genomes, displaying up to 100% nucleotide identity to their mobile counterparts. *L. adecarboxylata* is a rare human pathogen.

Tetracycline resistance genes

A *tetX* gene was identified in *Sphingobacterium* spp. strain PM2-P1-29[80]. Though the authors showed the gene to be located on a mobilizable transposon and noted its' similarity to conjugative transposon CTnDOT from *Bacteroides*, they conclude that *Sphingobacterium* spp. strain PM2-P1-29 may be the ancestral source of the *tetX* gene. Our analysis identified *tetX* in only 4 of 26 *Sphingobacterium spp.* In all of these, the *tetX*-locus contained several genes associated with mobility, such as *mob* genes, integrases and transposases. Other antibiotic resistance genes are identified at these loci as well, further indicating mobility. Due to the lack of non-mobile *tetX*-loci in any *Sphingobacterium* spp, there are no indications that *Sphingobacterium spp.* may be a recent origin of those genes.

Supplementary Table 1

Randomly selected non-origin proteobacterial species and origin species, information on association with infection in humans and animals

desulfovibrio litoralis	-	-	-	-	-	n
kingella potus	y	y	y	-	16000497	n
caballeronia humi	-	-	-	-	-	n
campylobacter geochelonis	-	y	-	-	27266587	n
mitsuaria chitosanitabida	-	-	-	-	-	n
sphingobium ummariense	-	-	-	-	-	n
pseudomonas straminea	-	-	-	-	-	n
corallincola platygyrae	-	-	-	-	-	n
dyella nitratireducens	-	-	-	-	-	n
azoarcus olearius	-	-	-	-	-	n
jannaschia helgolandensis	-	-	-	-	-	n
enterobacter chengduensis	y	-	y	n	30302649	n
altererythrobacter sediminis	-	-	-	-	-	n
corallococcus llansteffanensis	-	-	-	-	-	n
ancylobacter plantiphilus	-	-	-	-	-	n
thalassospira frigidphilosprofundus	-	-	-	-	-	n
psychromonas aquimarina	-	-	-	-	-	n
thermopetrobacter submarinus	-	-	-	-	-	n
methylorubrum zatmanii	y	-	y	-	9854105	n
aquisediminimonas sediminicola	-	-	-	-	-	n
oceanisphaera ostreae	-	-	-	-	-	n
parahaliea aestuarii	-	-	-	-	-	n
massilia yuzhufengensis	-	-	-	-	-	n
moraxella lacunata	y	y	y	y	3901656;30369588	n
shimia biformata	-	-	-	-	-	n
thiomonas arsenitoxydans	-	-	-	-	-	n
bradyrhizobium oligotrophicum	-	-	-	-	-	n
porphyrobacter meromictius	-	-	-	-	-	n
holospora caryophila	-	-	-	-	-	n
acinetobacter johnsonii	y	y	y	-	24600597	n
novosphingobium nitrogenifigens	-	-	-	-	-	n
glaciecola nitratireducens	-	-	-	-	-	n
tropicimonas arenosa	-	-	-	-	-	n
hirschia maritima	-	-	-	-	-	n
vibrio rhizosphaerae	-	-	-	-	-	n
photorhabdus noenieputensis	-	-	-	-	-	n
octadecabacter temperatus	-	-	-	-	-	n
providencia heimbachae	y	-	-	-	10449504	n
bradyrhizobium cytisi	-	-	-	-	-	n
roseivivax halotolerans	-	-	-	-	-	n

kaistia granuli	-	-	-	-	-	n
nitrosomonas marina	-	-	-	-	-	n
desulfovibrio oliviopondense	-	-	-	-	-	n
blastochloris viridis	-	-	-	-	-	n
rhodovulum salis	-	-	-	-	-	n
altererythrobacter deserti	-	-	-	-	-	n
acinetobacter gandensis	-	y	-	-	25225259	n
helicobacter brantae	-	y	-	-	16820454	n
alteromonas abrolhosensis	-	-	-	-	-	n
pseudopontivivens aestuariicola	-	-	-	-	-	n
chthonobacter albigriseus	-	-	-	-	-	n
photorhabdus luminescens	-	-	-	-	-	n
roseovarius aestuarii	-	-	-	-	-	n
desulfosarcina ovata	-	-	-	-	-	n
elioraea rosea	-	-	-	-	-	n
pasteurella mairii	-	y	-	y	15653877	n
cycloclasticus zancles	-	-	-	-	-	n
pseudooceanicola marinus	-	-	-	-	-	n
bowmanella denitrificans	-	y	-	-	29622614	n
erwinia aphidicola	-	-	-	-	-	n
yersinia aldovae	-	-	-	-	-	n
burkholderia australis	-	-	-	-	-	n
psychrobacter muriicola	-	-	-	-	-	n
neisseria oralis	y	-	y	-	22798652	n
steroidobacter denitrificans	y	-	-	-	10.4167/jbv.2014.44.3.244	n
bordetella bronchiseptica	y	y	y	y	32209128;1889042	n
pseudomonas rhodesiae	y	-	y	-	25278578	n
wenxinia marina	-	-	-	-	-	n
legionella bozemanii	y	-	y	-	24023988	n
xanthobacter viscosus	-	-	-	-	-	n
halomonas sabkhae	-	-	-	-	-	n
duganella ginsengisoli	-	-	-	-	-	n
sorangium cellulosum	-	-	-	-	-	n
bdellovibrio exovorus	-	-	-	-	-	n
ochrobactrum grignonense	-	-	-	-	-	n
rhodobium orientis	-	-	-	-	-	n
acidiphilium organovorum	-	-	-	-	-	n
caballeronia concitans	y	-	y	-	27375597	n
sphingomonas cynarae	-	-	-	-	-	n
acidiphilium acidophilum	-	-	-	-	-	n
bradyrhizobium ripae	-	-	-	-	-	n
novosphingobium colocasiae	-	-	-	-	-	n
chondromyces crocatus	-	-	-	-	-	n
neptuniibacter marinus	-	-	-	-	-	n
pseudorhodobacter aquimaris	-	-	-	-	-	n
celeribacter baekdonensis	-	-	-	-	-	n
desulfomicrobium baculatum	-	-	-	-	-	n
wenzhouxiangella sediminis	-	-	-	-	-	n

luteimonas vadosa	-	-	-	-	-	n
thalassobaculum litoreum	-	-	-	-	-	n
helicobacter fennelliae	y	y	y	-	27149471	n
thauera mechernichensis	-	-	-	-	-	n
rhodopseudomonas pseudopalustris	-	-	-	-	-	n
salinisphaera shabanensis	-	-	-	-	-	n
xenorhabdus bovienii	-	-	-	-	-	n
citrobacter freundii	y	y	y	y	PMC6505869	y
shewanella algae	y	y	y	-	30363620	y
enterobacter mori	y	-	y	-	10.1089/mdr.2018.0098	y
aeromonas caviae	y	y	y	y	10.1016/j.micpath.2017.07.031	y
aeromonas allosaccharophila	y	y	y	-	10.1093/jac/dkn341	y
aeromonas media	y	y	y	-	20065325	y
aeromonas sanarellii	y	-	y	-	10.1099/ijs.0.014621-0	y
morganella morganii	y	y	y	y	30353002	y
enterobacter asburiae	y	-	y	-	10.3928/01477447-20120525-52	y
enterobacter cloacae	y	y	y	y	10.1093/jac/dkw006	y
hafnia alvei	y	y	y	y	10.1080/09712119.2014.963086	y
klebsiella pneumoniae	y	y	y	y	10.1128/JCM.01537-18	y
kluyvera ascorbata	y	y	y	y	10.1292/jvms.08-0342	y
kluyvera georgiana	y	-	y	-	10.1093/jac/dks294	y
acinetobacter baumannii	y	y	y	y	21888812	y
acinetobacter guillouiae	y	-	y	-	25336457	y
moraxella pluranimalium	-	y	-	y	10.1099/ijs.0.006205-0	y
rheinheimera pacifica	-	-	-	-	-	y
leclercia adecarboxylata	y	y	y	-		y
acinetobacter gyllenbergii	y	-	y	-	26645270	y
acinetobacter radioresistens	y	y	y	y	10.1016/j.jiac.2017.03.011	y
shewanella xiamenensisi	y	y	y	-	10.1099/jmm.0.031625-0	y
salinivibrio socompensis	-	-	-	-	-	n

Supplementary table 1: columns from left to right: isolated from human, isolated from domestic animal, isolated from human infection site, isolated from animal infection site, reference (pubmed id or DOI), origin.

Supplementary References

1. Yoon, E.-J. *et al.* Origin in Acinetobacter guillouiae and dissemination of the aminoglycoside-modifying enzyme Aph(3')-VI. *MBio* **5**, e01972-14 (2014).

2. Yoon, E.-J. *et al.* Origin in *Acinetobacter gyllenbergii* and dissemination of aminoglycoside-modifying enzyme AAC(6′)-Ih. *J. Antimicrob. Chemother.* **71**, 601–606 (2016).

3. Barlow, M. & Hall, B. G. Origin and evolution of the AmpC beta-lactamases of Citrobacter freundii. *Antimicrob. Agents Chemother.* **46**, 1190–8 (2002).

4. Tzouvelekis, L. S. & Bonomo, R. A. SHV-type beta-lactamases. *Curr. Pharm. Des.* **5**, 847–64 (1999).

5. Haeggman, S., Löfdahl, S., Paauw, A., Verhoef, J. & Brisse, S. Diversity and evolution of the class A chromosomal beta-lactamase gene in Klebsiella pneumoniae. *Antimicrob. Agents Chemother.* **48**, 2400–8 (2004).

6. Ford, P. J. & Avison, M. B. Evolutionary mapping of the SHV -lactamase and evidence for two separate IS26-dependent blaSHV mobilization events from the Klebsiella pneumoniae chromosome. *J. Antimicrob. Chemother.* **54**, 69–75 (2004).

7. Haeggman, S., Löfdahl, S. & Burman, L. G. An allelic variant of the chromosomal gene for class A beta-lactamase K2, specific for Klebsiella pneumoniae, is the ancestor of SHV-1. *Antimicrob. Agents Chemother.* **41**, 2705–9 (1997).

8. Long, S. W. *et al.* Whole-Genome Sequencing of Human Clinical Klebsiella pneumoniae Isolates Reveals Misidentification and Misunderstandings of Klebsiella pneumoniae, Klebsiella variicola, and Klebsiella quasipneumoniae. *mSphere* **2**, (2017).

9. Cantón, R., González-Alba, J. M. & Galán, J. C. CTX-M Enzymes: Origin and Diffusion. *Front. Microbiol.* **3**, 110 (2012).

10. Poirel, L., Kämpfer, P. & Nordmann, P. Chromosome-encoded Ambler class A beta-lactamase of Kluyvera georgiana, a probable progenitor of a subgroup of CTX-M extended-spectrum beta-lactamases. *Antimicrob. Agents Chemother.* **46**, 4038–40

(2002).

11. Olson, A. B. *et al.* Identification of a progenitor of the CTX-M-9 group of extended-spectrum beta-lactamases from Kluyvera georgiana isolated in Guyana. *Antimicrob. Agents Chemother.* **49**, 2112–5 (2005).

12. Humeniuk, C. *et al.* Beta-lactamases of Kluyvera ascorbata, probable progenitors of some plasmid-encoded CTX-M types. *Antimicrob. Agents Chemother.* **46**, 3045–9 (2002).

13. Lartigue, M.-F., Poirel, L., Aubert, D. & Nordmann, P. In vitro analysis of ISEcp1B-mediated mobilization of naturally occurring beta-lactamase gene blaCTX-M of Kluyvera ascorbata. *Antimicrob. Agents Chemother.* **50**, 1282–6 (2006).

14. Cantón, R., González-Alba, J. M. & Galán, J. C. CTX-M Enzymes: Origin and Diffusion. *Front. Microbiol.* **3**, 110 (2012).

15. Ebmeyer, S., Kristiansson, E. & Larsson, D. G. J. PER extended-spectrum β-lactamases originate from Pararheinheimera spp. *Int. J. Antimicrob. Agents* (2018) doi:10.1016/J.IJANTIMICAG.2018.10.019.

16. Wu, J., Xie, L., Zhang, F., Ni, Y. & Sun, J. Molecular characterization of ISCR1-mediated blaPER-1 in a non-O1, non-O139 Vibrio cholerae strain from China. *Antimicrob. Agents Chemother.* **59**, 4293–5 (2015).

17. Mancini, S., Poirel, L., Kieffer, N. & Nordmann, P. Transposition of Tn1213 Encoding the PER-1 Extended-Spectrum β-Lactamase. *Antimicrob. Agents Chemother.* **62**, e02453-17 (2018).

18. Gupta, H. K., Gupta, R. D., Singh, A., Chauhan, N. S. & Sharma, R. Genome sequence of Rheinheimera sp. strain A13L, isolated from Pangong Lake, India. *J. Bacteriol.* **193**, 5873–4 (2011).

19. Sisinthy, S., Chakraborty, D., Adicherla, H. & Gundlapally, S. R. Emended description of the family Chromatiaceae, phylogenetic analyses of the genera Alishewanella, Rheinheimera and Arsukibacterium, transfer of Rheinheimera longhuensis LH2-2T to the genus Alishewanella and description of Alishewanella alkalitolerans sp. *Antonie Van Leeuwenhoek* **110**, 1227–1241 (2017).

20. Gudeta, D. D. *et al. Chromobacterium* spp. harbour Ambler class A β-lactamases showing high identity with KPC. *J. Antimicrob. Chemother.* **71**, 1493–1496 (2016).

21. Schauer, J., Gatermann, S. G., Hoffman, D., Lars, H. & Pfennigwerth, N. GPC-1, a novel class A carbapenemase detected in a clinical Pseudomonas aeruginosa isolate | Journal of Antimicrobial Chemotherapy | Oxford Academic. *J. Antimicrob. Chemother.* **75**, 911–916 (2020).

22. Nicoletti, A. G. *et al.* Characterization of BKC-1 class a carbapenemase from Klebsiella pneumoniae clinical isolates in Brazil. *Antimicrob. Agents Chemother.* **59**, 5159–5164 (2015).

23. Kieffer, N., Ebmeyer, S. & Larsson, D. G. J. The class A carbapanemases BKC-1 and GPC-1 both originate from the bacterial genus Shinella . *Antimicrob. Agents Chemother.* **64**, (2020).

24. Lange, F. *et al.* LMB-1, a novel family of class B3 MBLs from an isolate of Enterobacter cloacae. *J. Antimicrob. Chemother.* **73**, 2331–2335 (2018).

25. EBMEYER, S., KRISTIANSSON, E. & LARSSON, D. G. J. The mobile FOX AmpC beta-lactamases originated in Aeromonas allosaccharophila. *Int. J. Antimicrob. Agents* (2019) doi:10.1016/J.IJANTIMICAG.2019.09.017.

26. Ebmeyer, S., Kristiansson, E. & Larsson, D. G. J. CMY-1/MOX-family AmpC β-lactamases MOX-1, MOX-2 and MOX-9 were mobilized independently from three *Aeromonas* species. *J. Antimicrob. Chemother.* (2019) doi:10.1093/jac/dkz025.

27. Lallement, C., Pasternak, C., Ploy, M.-C. & Jové, T. The Role of ISCR1-Borne POUT Promoters in the Expression of Antibiotic Resistance Genes. *Front. Microbiol.* **9**, 2579 (2018).

28. Janda, J. M. & Abbott, S. L. The genus Aeromonas: taxonomy, pathogenicity, and infection. *Clin. Microbiol. Rev.* **23**, 35–73 (2010).

29. Batra, P., Mathur, P. & Misra, M. C. Aeromonas spp.: An Emerging Nosocomial Pathogen. *J. Lab. Physicians* **8**, 1–4 (2016).

30. Pietsch, M. *et al.* Whole genome analyses of CMY-2-producing Escherichia coli isolates from humans, animals and food in Germany. *BMC Genomics* **19**, 601 (2018).

31. Wu, S. W., Dornbusch, K., Kronvall, G. & Norgren, M. Characterization and nucleotide sequence of a Klebsiella oxytoca cryptic plasmid encoding a CMY-type beta-lactamase: confirmation that the plasmid-mediated cephamycinase originated from the Citrobacter freundii AmpC beta-lactamase. *Antimicrob. Agents Chemother.* **43**, 1350–7 (1999).

32. Fang, L.-X. *et al.* ISEcp1-mediated transposition of chromosome-borne blaCMY-2 into an endogenous ColE1-like plasmid in Escherichia coli. *Infect. Drug Resist.* **11**, 995–1005 (2018).

33. Barnaud, G. *et al.* Salmonella enteritidis: AmpC plasmid-mediated inducible beta-lactamase (DHA-1) with an ampR gene from Morganella morganii. *Antimicrob. Agents Chemother.* **42**, 2352–8 (1998).

34. Hsieh, W.-S., Wang, N.-Y., Feng, J.-A., Weng, L.-C. & Wu, H.-H. Identification of DHA-23, a novel plasmid-mediated and inducible AmpC beta-lactamase from Enterobacteriaceae in Northern Taiwan. *Front. Microbiol.* **6**, 436 (2015).

35. Fortineau, N., Poirel, L. & Nordmann, P. Plasmid-mediated and inducible cephalosporinase DHA-2 from Klebsiella pneumoniae. *J. Antimicrob. Chemother.* **47**,

207–210 (2001).

36. Liu, H., Zhu, J., Hu, Q. & Rao, X. Morganella morganii, a non-negligent opportunistic pathogen. *Int. J. Infect. Dis.* **50**, 10–17 (2016).

37. Jacoby, G. A. AmpC beta-lactamases. *Clin. Microbiol. Rev.* **22**, 161–82, Table of Contents (2009).

38. Rottman, M. *et al.* Chromosomal *ampC* genes in *Enterobacter* species other than *Enterobacter cloacae* , and ancestral association of the ACT-1 plasmid-encoded cephalosporinase to *Enterobacter asburiae*. *FEMS Microbiol. Lett.* **210**, 87–92 (2002).

39. Morand, P. C. *et al.* Specific distribution within the Enterobacter cloacae complex of strains isolated from infected orthopedic implants. *J. Clin. Microbiol.* **47**, 2489–95 (2009).

40. Kang, C.-I. *et al.* Bloodstream Infections Caused by Enterobacter Species: Predictors of 30-Day Mortality Rate and Impact of Broad-Spectrum Cephalosporin Resistance on Outcome. *Clin. Infect. Dis.* **39**, 812–818 (2004).

41. Papanicolaou, G. A., Medeiros, A. A. & Jacoby, G. A. Novel plasmid-mediated beta-lactamase (MIR-1) conferring resistance to oxyimino- and alpha-methoxy beta-lactams in clinical isolates of Klebsiella pneumoniae. *Antimicrob. Agents Chemother.* **34**, 2200–9 (1990).

42. Jacoby, G. A. & Tran, J. Sequence of the MIR-1 β-Lactamase Gene. *Antimicrob. Agents Chemother.* **43**, 1759–1760 (1999).

43. Conceição, T. *et al.* New chromosomal AmpC beta-lactamase in Enterobacter cloacae. *Antimicrob. Agents Chemother.* **48**, 1437 (2004).

44. Reisbig, M. D. & Hanson, N. D. Promoter sequences necessary for high-level expression of the plasmid-associated ampC beta-lactamase gene blaMIR-1.

Antimicrob. Agents Chemother. **48**, 4177–82 (2004).

45. Nadjar, D. *et al.* Outbreak of Klebsiella pneumoniae producing transferable AmpC-type beta-lactamase (ACC-1) originating from Hafnia alvei. *FEMS Microbiol. Lett.* **187**, 35–40 (2000).

46. Papagiannitsis, C. C., Tzouvelekis, L. S., Tzelepi, E. & Miriagou, V. Plasmid-encoded ACC-4, an extended-spectrum cephalosporinase variant from Escherichia coli. *Antimicrob. Agents Chemother.* **51**, 3763–7 (2007).

47. Poirel, L., Decousser, J.-W. & Nordmann, P. Insertion Sequence ISEcp1B Is Involved in Expression and Mobilization of a blaCTX-M β-Lactamase Gene. *Antimicrob. Agents Chemother.* **47**, 2938–2945 (2003).

48. Litrenta, J. & Oetgen, M. Hafnia alvei: A new pathogen in open fractures. *Trauma case reports* **8**, 41–45 (2017).

49. Poirel, L., Figueiredo, S., Cattoir, V., Carattoli, A. & Nordmann, P. Acinetobacter radioresistens as a silent source of carbapenem resistance for Acinetobacter spp. *Antimicrob. Agents Chemother.* **52**, 1252–6 (2008).

50. Corvec, S., Poirel, L., Naas, T., Drugeon, H. & Nordmann, P. Genetics and expression of the carbapenem-hydrolyzing oxacillinase gene blaOXA-23 in Acinetobacter baumannii. *Antimicrob. Agents Chemother.* **51**, 1530–3 (2007).

51. Wang, H. *et al.* Molecular epidemiology of clinical isolates of carbapenem-resistant Acinetobacter spp. from Chinese hospitals. *Antimicrob. Agents Chemother.* **51**, 4022–8 (2007).

52. Potron, A., Poirel, L. & Nordmann, P. Origin of OXA-181, an emerging carbapenem-hydrolyzing oxacillinase, as a chromosomal gene in Shewanella xiamenensis. *Antimicrob. Agents Chemother.* **55**, 4405–7 (2011).

53. Potron, A. *et al.* Characterization of OXA-181, a carbapenem-hydrolyzing class D beta-lactamase from Klebsiella pneumoniae. *Antimicrob. Agents Chemother.* **55**, 4896–9 (2011).

54. Tacão, M., Araújo, S., Vendas, M., Alves, A. & Henriques, I. Shewanella species as the origin of blaOXA-48 genes: insights into gene diversity, associated phenotypes and possible transfer mechanisms. *Int. J. Antimicrob. Agents* **51**, 340–348 (2018).

55. Evans, B. A., Hamouda, A., Towner, K. J. & Amyes, S. G. B. OXA-51-like β-lactamases and their association with particular epidemic lineages of Acinetobacter baumannii. *Clin. Microbiol. Infect.* **14**, 268–275 (2008).

56. Chen, T.-L. *et al.* Emergence and Distribution of Plasmids Bearing the blaOXA-51-like gene with an upstream ISAba1 in carbapenem-resistant Acinetobacter baumannii isolates in Taiwan. *Antimicrob. Agents Chemother.* **54**, 4575–81 (2010).

57. Kieffer, N., Nordmann, P. & Poirel, L. Moraxella Species as Potential Sources of MCR-Like Polymyxin Resistance Determinants. *Antimicrob. Agents Chemother.* **61**, AAC.00129-17 (2017).

58. Sun, J. *et al.* Deciphering MCR-2 Colistin Resistance. *MBio* **8**, (2017).

59. Yin, W. *et al.* Novel Plasmid-Mediated Colistin Resistance Gene mcr-3 in Escherichia coli. *MBio* **8**, e00543-17 (2017).

60. Shen, Y. *et al.* Prevalence and Genetic Analysis of mcr-3-Positive Aeromonas Species from Humans, Retail Meat, and Environmental Water Samples. *Antimicrob. Agents Chemother.* **62**, e00404-18 (2018).

61. Zhang, H. *et al.* Action and mechanism of the colistin resistance enzyme MCR-4. *Commun. Biol.* **2**, 36 (2019).

62. Carattoli, A. *et al.* Novel plasmid-mediated colistin resistance mcr-4 g ene in

Salmonella and Escherichia coli , Italy 2013, Spain and Belgium, 2015 to 2016. *Eurosurveillance* **22**, 30589 (2017).

63. Khedher, M. Ben *et al.* Massive analysis of 64'628 bacterial genomes to decipher a water reservoir and origin of mobile colistin resistance (mcr) gene variants: is there another role for this family of enzymes? *bioRxiv* 763474 (2019) doi:10.1101/763474.

64. Kieffer, N. *et al.* mcr-9, an Inducible Gene Encoding an Acquired Phosphoethanolamine Transferase in Escherichia coli, and Its Origin With correction see https://aac.asm.org/content/63/11/e01866-19. doi:10.1128/AAC.00965-19.

65. Poirel, L., Rodriguez-Martinez, J.-M., Mammeri, H., Liard, A. & Nordmann, P. Origin of plasmid-mediated quinolone resistance determinant QnrA. *Antimicrob. Agents Chemother.* **49**, 3523–5 (2005).

66. Melvold, J. A. *et al.* Identification of a novel qnrA allele, qnrA8, in environmental Shewanella algae. *J. Antimicrob. Chemother.* **72**, 2949–2952 (2017).

67. Tseng, S.-Y. *et al.* The Pathogenicity of *Shewanella algae* and Ability to Tolerate a Wide Range of Temperatures and Salinities. *Can. J. Infect. Dis. Med. Microbiol.* **2018**, 1–9 (2018).

68. Jacoby, G. A., Griffin, C. M. & Hooper, D. C. Citrobacter spp. as a source of qnrB Alleles. *Antimicrob. Agents Chemother.* **55**, 4979–84 (2011).

69. Ribeiro, T. G., Novais, Â., Branquinho, R., Machado, E. & Peixe, L. Phylogeny and Comparative Genomics Unveil Independent Diversification Trajectories of qnrB and Genetic Platforms within Particular Citrobacter Species. *Antimicrob. Agents Chemother.* **59**, 5951–8 (2015).

70. Liu, L. *et al.* Genetic Diversity, Multidrug Resistance, and Virulence of Citrobacter freundii From Diarrheal Patients and Healthy Individuals. *Front. Cell. Infect. Microbiol.* **8**, 233 (2018).

71. Da Re, S. *et al.* The SOS response promotes qnrB quinolone-resistance determinant expression. *EMBO Rep.* **10**, 929–33 (2009).

72. Cattoir, V., Nordmann, P., Silva-Sanchez, J., Espinal, P. & Poirel, L. ISEcp1-mediated transposition of qnrB-like gene in Escherichia coli. *Antimicrob. Agents Chemother.* **52**, 2929–32 (2008).

73. Rezaei, M., Akya, A., Elahi, A., Ghadiri, K. & Jafari, S. The clonal relationship among the Citrobacter freundii isolated from the main hospital in Kermanshah, west of Iran. *Iran. J. Microbiol.* **8**, 175–180 (2016).

74. Albornoz, E. *et al.* qnrE1, a Member of a New Family of Plasmid-Located Quinolone Resistance Genes, Originated from the Chromosome of Enterobacter Species. *Antimicrob. Agents Chemother.* **61**, e02555-16 (2017).

75. Kim, H. Bin *et al.* oqxAB encoding a multidrug efflux pump in human clinical isolates of Enterobacteriaceae. *Antimicrob. Agents Chemother.* **53**, 3582–4 (2009).

76. Ito, R. *et al.* Widespread Fosfomycin Resistance in Gram-Negative Bacteria Attributable to the Chromosomal fosA Gene. *MBio* **8**, (2017).

77. Ito, R., Pacey, M. P., Mettus, R. T., Sluis-Cremer, N. & Doi, Y. Origin of the plasmid-mediated fosfomycin resistance gene fosA3. *J. Antimicrob. Chemother.* **73**, 373–376 (2018).

78. Rodriguez, M. M., Ghiglione, B., Power, P., Naas, T. & Gutkind, G. Proposing Kluyvera georgiana as the Origin of the Plasmid-Mediated Resistance Gene fosA4. *Antimicrob. Agents Chemother.* **62**, e00710-18 (2018).

79. Poirel, L. *et al.* Identification of FosA8, a Plasmid-Encoded Fosfomycin Resistance Determinant from Escherichia coli, and Its Origin in Leclercia adecarboxylata. *Antimicrob. Agents Chemother.* **63**, (2019).

80. Ghosh, S., Sadowsky, M. J., Roberts, M. C., Gralnick, J. A. & LaPara, T. M. *Sphingobacterium* sp. strain PM2-P1-29 harbours a functional *tet* (X) gene encoding for the degradation of tetracycline. *J. Appl. Microbiol.* **106**, 1336–1342 (2009).
