# Supplementary material for: A framework for identifying the recent origins of mobile antibiotic resistance genes
Source: Commun Biol. 2021 Jan 4;4:8. doi: 10.1038/s42003-020-01545-5 (PMC7782503; doi:10.1038/s42003-020-01545-5)
Supplement: Supplementary file 3 — Reporting Summary [file 42003_2020_1545_MOESM3_ESM.pdf]

nature research

Corresponding author(s): D.G Joakim Larsson

Last updated by author(s): Oct 14, 2020

Reporting Summary

Nature Research wishes to improve the reproducibility of the work that we publish. This form provides structure for consistency and transparency in reporting. For further information on Nature Research policies, see our Editorial Policies and the Editorial Policy Checklist.

Statistics

For all statistical analyses, confirm that the following items are present in the figure legend, table legend, main text, or Methods section.

n/a	Confirmed	
☐	☒	The exact sample size (*n*) for each experimental group/condition, given as a discrete number and unit of measurement
☐	☒	A statement on whether measurements were taken from distinct samples or whether the same sample was measured repeatedly
☐	☒	The statistical test(s) used AND whether they are one- or two-sided<br>*Only common tests should be described solely by name; describe more complex techniques in the Methods section.*
☒	☐	A description of all covariates tested
☒	☐	A description of any assumptions or corrections, such as tests of normality and adjustment for multiple comparisons
☒	☐	A full description of the statistical parameters including central tendency (e.g. means) or other basic estimates (e.g. regression coefficient) AND variation (e.g. standard deviation) or associated estimates of uncertainty (e.g. confidence intervals)
☐	☒	For null hypothesis testing, the test statistic (e.g. *F*, *t*, *r*) with confidence intervals, effect sizes, degrees of freedom and *P* value noted<br>*Give P values as exact values whenever suitable.*
☒	☐	For Bayesian analysis, information on the choice of priors and Markov chain Monte Carlo settings
☒	☐	For hierarchical and complex designs, identification of the appropriate level for tests and full reporting of outcomes
☒	☐	Estimates of effect sizes (e.g. Cohen's *d*, Pearson's *r*), indicating how they were calculated

Our web collection on statistics for biologists contains articles on many of the points above.

Software and code

Policy information about availability of computer code

Data collection	Custom code was used for data collection. The GenBank bacterial assembly summary file was downloaded from ftp://ftp.ncbi.nlm.nih.gov/genomes/genbank/bacteria/assembly_summary.txt and the urls for each assembly was extracted. For plasmids, accession numbers were downloaded from ftp://ftp.ncbi.nlm.nih.gov/refseq/release/plasmid/. 'wget' was then used to download all assembly/ plasmid sequences
Data analysis	ARG identification: CARD, ResFinder(downloaded September 2019, novel ARGs LMB-1, FosA8, FosL and GPC-1 were manually added), DIAMOND v0.9.24.125 (70% cutoff); Gene prediction and annotation: Prodigal v2.6.3, Uniprot KB (downloaded January 2019); Integron identification: IntegronFinder v2; Manual IS identification: https://isfinder.biotoul.fr/ ; Alignment and phylogeny: MAFFT v7.310, FastTree v2.1.11;clustering: USEARCH v8.0.1445. Custom code: File handling, pipeline creation, visualization of genetic context in python 2.7/3.7 Custom code is available at https://github.com/EbmeyerSt/ARG_loci_comparative_pipeline

For manuscripts utilizing custom algorithms or software that are central to the research but not yet described in published literature, software must be made available to editors and reviewers. We strongly encourage code deposition in a community repository (e.g. GitHub). See the Nature Research guidelines for submitting code & software for further information.

Data

Policy information about availability of data

All manuscripts must include a data availability statement. This statement should provide the following information, where applicable:
- Accession codes, unique identifiers, or web links for publicly available datasets
- A list of figures that have associated raw data
- A description of any restrictions on data availability

Figures with associated raw data: Figure 4, raw data provided in supplementary file 2. Accession codes for individual genomes are provided in the respective study (if applicable), referenced in supplementary

file 1. Due to the large number of genomes analyzed in this study, we do not provide accession numbers for every genome, but can provide them upon request. Please note that this manuscript does not involve any generation of new DNA sequence data. We just refer to already published sequences.

Field-specific reporting

Please select the one below that is the best fit for your research. If you are not sure, read the appropriate sections before making your selection.

☐ Life sciences ☐ Behavioural & social sciences ☒ Ecological, evolutionary & environmental sciences

For a reference copy of the document with all sections, see nature.com/documents/nr-reporting-summary-flat.pdf

Ecological, evolutionary & environmental sciences study design

All studies must disclose on these points even when the disclosure is negative.

Study description	Literature study on proposed origins of resistance genes. A set of formalized criteria was formulated and used to scrutinize all proposed origins using data available in the literature and a comparative genomics approach based on a compilation of available DNA sequence data. Patterns in curated origins were analyzed.
Research sample	All available genome assemblies and plasmids from GenBank containing the respective resistance gene
Sampling strategy	All genomes and plasmids containing a respective resistance gene (or very similar sequence) were included in the comparison
Data collection	Download of all available sequenced genome assemblies and plasmids from GenBank
Timing and spatial scale	All data were downloaded February 2020. All available assemblies and plasmids were downloaded from GenBank
Data exclusions	Assembly or plasmid sequences containing less than 6 genes in the genetic environment were excluded from the comparison. This was done to reduce redundancy in the visualization step (as resistance genes on such short contigs are usually mobile. Mobile genes however are also present on complete plasmids, and these sequences are more informative). Even if such sequences were not obviously mobile, the information that can be gained from this short sequence is insufficient to conclude that the respective gene is not mobile
Reproducibility	All steps of the analysis are described in detail in the materials and methods part. All analyzed genomes and used code are publicly available. The<br>analysis was repeated several times during the writing of the manuscript, with the results always being reproducible. But please note that this is not an experimental study, so it is in a way questionable if reproducibility is an aspect that is applicable here.
Randomization	Not relevant, all genomes containing a resistance gene were analyzed
Blinding	Not relevant, all genomes containing a resistance gene were analyzed, irrespective of previous reports

Did the study involve field work? ☐ Yes ☒ No

Reporting for specific materials, systems and methods

We require information from authors about some types of materials, experimental systems and methods used in many studies. Here, indicate whether each material, system or method listed is relevant to your study. If you are not sure if a list item applies to your research, read the appropriate section before selecting a response.

Materials & experimental systems

n/a	Involved in the study	
☒	☐	Antibodies
☒	☐	Eukaryotic cell lines
☒	☐	Palaeontology and archaeology
☒	☐	Animals and other organisms
☒	☐	Human research participants
☒	☐	Clinical data
☒	☐	Dual use research of concern

Methods

n/a	Involved in the study	
☒	☐	ChIP-seq
☒	☐	Flow cytometry
☒	☐	MRI-based neuroimaging
